# Bite force–gape curves and passive tension costs in *Macaca mulatta*

**DOI:** 10.1242/jeb.251950

**Published:** 2026-05-05

**Authors:** Stephanie L. Canington, Carla Escabi, Michael L. Platt, Timothy A. Machado, Jose Iriarte-Diaz, Myra F. Laird

**Affiliations:** ^1^ Department of Basic and Translational Sciences, University of Pennsylvania School of Dental Medicine, Philadelphia, PA 19104, USA; ^2^ Department of Neuroscience, Perelman School of Medicine, University of Pennsylvania, Philadelphia, PA 19104, USA; ^3^ Department of Psychology, School of Arts and Sciences, University of Pennsylvania, Philadelphia, PA 19104, USA; ^4^ Marketing Department, Wharton School of Business, University of Pennsylvania, Philadelphia, PA 19104, USA; ^5^ Howard Hughes Medical Institute, University of Pennsylvania Perelman School of Medicine, Philadelphia, PA 19104, USA; ^6^ Department of Biology, University of the South, Sewanee, TN 37383, USA

**Keywords:** Muscle, Feeding, Mastication, Primate, Dental

## Abstract

Passive forces generated by the jaw adductor muscles and their connective tissues are thought to play a protective role in the feeding system by limiting gape to avoid hyperextension and minimize distractive forces at the temporomandibular joint. However, passive muscle forces have only been measured in individual jaw adductors of two non-primate mammals, and it is unknown how these forces translate to bite force at the occlusal surface and affect gape behaviors. We measured *in vivo* passive bite forces in eight adult *Macaca mulatta* at anterior (I_1_) and posterior (M_1_) bite points across linear gapes ranging from 15 to 50 mm. Active bite force data were collected at the anterior bite point from two of these macaques (one male, one female) using a custom-built bite force transducer across linear gapes ranging from 10 to 60 mm. We demonstrate that *M. mulatta* passive bite forces increase with gape and vary by bite point, with forces larger at M_1_ compared with I_1_ for both linear and angular gapes. Our experimental data and Hill-type muscle models of both active and passive forces suggest that passive bite forces are absolutely and relatively small at the occlusal surface in macaques and play a minimal role in constraining gape. These are the first empirical data on bite force passive tension in primates, and the first data to suggest that the macaque jaw adductor muscles exhibit unusually high compliance, potentially relating to selection for large gape behaviors.

## Teaser

**Summary:** This study compares *in vivo* passive and active bite forces at the occlusal surface in *Macaca mulatta* and discusses implications for bite force–gape tradeoffs and feeding system muscle models.

## INTRODUCTION

**Fig. 1.**
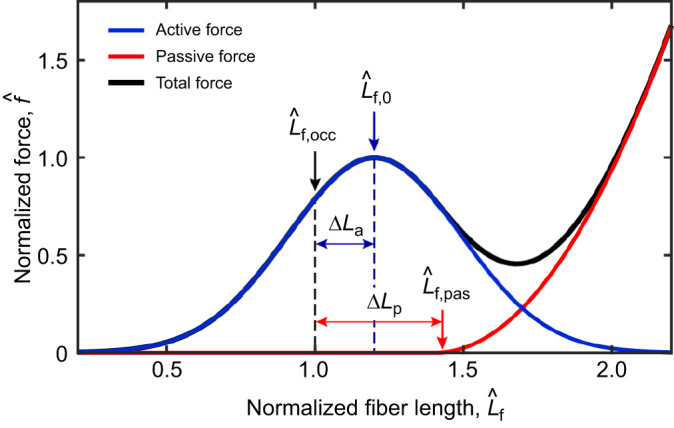
**A theoretical muscle force–length curve.** Fiber length is normalized to 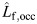, the fiber length at occlusion. Active force increases until the fiber reaches an optimum length, 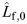, after which active tension decreases. Passive force exponentially increases after it reaches the slack length, 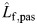. Δ*L*_a_ and Δ*L*_p_ are the active and passive offset parameters, respectively, that indicate the difference in normalized fiber length.

Bite force is a product of integrated neural signaling, skeletal biomechanics and muscle physiology. In mammals, bite force is primarily generated by the three major jaw adductor muscles: the masseter, the medial pterygoid and the temporalis. The total force generated by each of these muscles is the result of both active forces produced by the cross-bridging cycles within sarcomeres (Gordon et al., 1966) and passive tensile forces generated by connective tissue elements (Purslow, 2020), intracellular proteins such as titin (Labeit and Kolmerer, 1995), and the intermediate filament system (Lazarides, 1980). Contractile properties at the level of the sarcomere, the smallest functional contractile unit, as well as the whole muscle (Winters et al., 2011), can be described as a length–tension curve, where force increases until the sarcomere or muscle reaches an optimal point of stretch (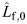, the optimal normalized fiber length). If stretched beyond this point, the active force decreases and the passive force increases (Fig. 1). Length–tension curves and the role of passive tension have primarily been studied in isolated muscle preparations (e.g. Davis et al., 2003). Applying these findings to broader functional units, such as the jaw or leg, is challenging because the muscles are a part of complex, integrated systems.

Passive tension is thought to contribute to the mammalian feeding system in several ways. Jaw adductor passive tension was initially proposed to help maintain a resting closed to slightly open jaw position (Yemm, 1976; Rugh et al., 1980; Miller, 1991), but Langenbach and Hannam (1999) suggest both passive and active tension help to maintain jaw position. In the locomotor system, passive tension provides elastic energy storage in muscles such as the gastrocnemius, contributing to energy efficiency during locomotion (Biewener, 1998; Ettema, 1996). However, energy costs of the feeding system are small (Laird et al., 2016; van Casteren et al., 2022; Wall et al., 2023), as is the likely importance of passive tension in energy storage for the jaw (Ross and Iriarte-Diaz, 2019). Instead, passive tension is thought to play a protective role in the feeding system by limiting gape to avoid hyperextension, sudden lengthening perturbations, and by minimizing distractive forces at the temporomandibular joint (TMJ; Iriarte-Diaz et al., 2026). This protective mechanism has been demonstrated by Horner and colleagues (2024), who showed that the medial gastrocnemius of rats increases in passive stiffness as the operating length of the muscle shortens, likely limiting the force the animal produces during locomotion. In cercopithecoid primates, passive tension safeguards against TMJ displacement during wide-gape behaviors, including displays, prey capture and food ingestion. For example, the wide-gaped canine display in species such as macaques relates to inter-male aggression and an increased capacity for adductor muscle stretch (Plavcan and van Schaik, 1992; Taylor et al., 2024).

There are few assessments of both active and passive tension in the mammalian feeding muscles. Both active and passive tension were modeled in gouging marmosets and non-gouging tamarins using passive tension data based on the tibialis anterior of a rabbit, and tamarins had larger passive forces at wide gapes (Eng et al., 2009). Anapol and Herring (1989) reported active and passive tension in the masseter and digastric muscles of miniature pigs using nerve cuff stimulation. They found that masseter passive tension costs began to increase prior to the gape associated with optimal force production. Similar results were noted in the masticatory muscles of opossums, which have a larger relative maximum gape (Thexton and Hiiemae, 1975), as well as in rats, where the superficial masseter is proposed to operate at both long and likely unstable lengths while biting mechanically hard foods (Konow et al., 2025). However, more recent analyses of active and passive tension measured in isolated limb muscles suggest that passive tension is dependent upon the connective tissue of the individual muscle and does not consistently scale with active tension (Winters et al., 2011; Ward et al., 2020; Lieber et al., 2025).

The effects of jaw adductor active and passive tension on bite force at the occlusal surface are of interest for musculoskeletal models of the feeding system. Hill-type muscle models use computational approaches to functional systems that incorporate bony geometry with muscle properties, including muscle architecture measures of fiber length, pinnation angle and passive tension. While these models are commonly used for the locomotor system (e.g. Hill, 1938, 1950; Anderson and Pandy, 2003; Thelen, 2003; Delp et al., 2007; Peterson et al., 2011; Biewener et al., 2014), such models have only recently been applied to the feeding system (Iriarte-Diaz et al., 2017, 2026; Laird et al., 2024). Muscle models of bite force were compared with *in vivo* bite forces from two strepsirrhine primates, *Eulemur* and *Varecia*, and modeled passive components had little to no effect on predicting bite force (Laird et al., 2024). However, it is unknown how modeled and *in vivo* occlusal passive forces compare in primates.

We compared total and passive bite forces at the occlusal surface of rhesus macaques (*Macaca mulatta*) and tested the effects of passive tension on muscle models of the feeding system. To estimate the contribution of both active and passive components of muscle force to bite force, we measured bite forces from awake and sedated animals, respectively. Bite forces obtained from sedated animals represent only the passive bite forces. Bite forces recorded from awake animals represent the total bite forces, which combine both passive and active muscle force contributions. Macaques are an ideal model organism for comparing jaw adductor active and passive muscle force as they use both high bite force and large gapes to orally process foods, to engage in aggressive or defensive exchanges, and for social or manipulative purposes (e.g. Corlett and Lucas, 1990; Hylander, 2013; Panagiotopoulou et al., 2023). Bite forces in macaques are well studied, with estimates derived from numerous methods including *in vivo*, muscle, tooth mark and simulation approaches (Laird et al., 2025). Previous *in vivo* approaches to bite force in macaques report a range of 92.18 to 101.99 N on the incisors and an average of 235 N (maximum 333 N) on the molars using a force transducer (Hylander, 1979). Dechow and Carlson (1990) used unilateral muscle stimulation to record a mean range of 133.1 to 151.1 N on the incisors and 286.2 to 369.3 N on the molars in a sample of 96 adult *M. mulatta*. However, there are no published *in vivo* passive bite force values for the primate feeding system, with models including passive muscle forces that have been estimated using data either from other animals (Thexton and Hiiemae, 1975; Anapol and Herring, 1989; van Eijden et al., 2002) or from muscles outside the feeding system (e.g. rabbit tibialis anterior; Davis et al., 2003). Here, we addressed three questions on the role of passive bite forces.

### Question 1: how do passive bite forces relate to gape and vary across the toothrow?

As the jaw is gaped and adductor muscles are stretched, passive muscle and bite forces are expected to increase exponentially. Passive bite forces are expected to vary along the toothrow such that posterior bite points are associated with higher bite force values, reflecting the proximity of the bite point to the TMJ (Greaves, 1978).

### Question 2: how do passive bite forces relate to peak active bite force and maximum gape?

Masticatory muscle passive tension data from minipigs suggest passive tension costs rise prior to the gape associated with peak bite force, and passive tension values exceed active tension values at or around maximum gape. This serves to safeguard against jaw displacement at the expense of bite force production (Thexton and Hiiemae, 1975; Anapol and Herring, 1989). We expected macaques to show a similar pattern, with passive bite force values rising prior to peak active bite force and surpassing active tension values near maximum gape.

### Question 3: how do passive forces influence Hill-type muscle model predictions of bite force and operating range?

Models of the primate feeding muscles either do not include passive muscle forces or estimate passive tension from other animals and/or muscles (e.g. Thexton and Hiiemae, 1975; Anapol and Herring, 1989; Davis et al., 2003). We expected the inclusion of passive muscle force estimates from *in vivo* passive bite force data in Hill-type models of the feeding system to produce similar bite force and operating range estimates to models without passive muscle force estimates (Laird et al., 2024). However, we expected bite force and operating range estimates to be different from models using passive tension estimates from other animals and/or muscles.

## MATERIALS AND METHODS

### Passive and active tension data

A total of 74 measures of *in vivo* passive bite force were collected from eight *Macaca mulatta* (Zimmermann 1780) (5 females, 3 males) adults at linear gapes ranging from 15 to 50 mm. Total bite force–gape data were collected from two of the macaques included in the passive bite force sample (HK male and LL female). Active bite force–gape data were not collected for the other six macaques in the passive sample because of animal training availability. All data were collected at the University of Pennsylvania and approved by the University of Pennsylvania Institutional Animal Care and Use Committee (#805870).

Bite force data were recorded using a custom-built bite force transducer based on a model described by Herrel and colleagues (2001, 2004, 2005) and others (e.g. Verwaijen et al., 2002; Aguirre et al., 2003; Laird et al., 2023a, 2024). Two metal plates were fixed to a compressive piezoelectric load cell (Kistler 9203), and the spacing between the plates was changed using an adjustable micrometer (Mitutoyo 152-103). Increased space between the bite plates resulted in a larger linear gape (in mm). Compressive loads were amplified (Kistler 5995A), passed through an analog-to-digital converter (Adafruit Industries ADS1115), and logged on a Raspberry Pi 4 Model B using custom Python code. Athletic tape was wrapped around the metal plates to protect the animal's teeth. Amplified output loads were converted to newtons of force by conducting calibration experiments using a series of 100, 200 and 500 g weights that were statically placed on the bite plates, including the athletic tape, at 10, 15 and 20 mm linear gape determined by spacing between the bite plates. There were no significant differences in the forces between gapes in the calibration experiments, and we used a standard correction factor (Laird et al., 2023a, 2024).

Passive bite force data were collected using this bite force transducer during semi-annual veterinary sedations conducted by University Laboratory Animal Resources veterinarians and veterinary technicians in February 2024 and 2025. Sedation was achieved using a combination of 4 mg kg^−1^ ketamine and 0.025 mg kg^−1^ dexmedetomidine, and all animals had a negative palpebral reflex and relaxed jaw tone before passive bite force data collection. While the animal was sedated, the bite plates of the transducer were successively spaced between 15 and 50 mm linear gape in 5 mm increments and positioned between the central incisors and between the right first molars. The bite plates were held between the teeth until the force plateaued; the bite plates were then removed and the jaw was allowed to close between each gape increment and between bite positions on the incisors and molars. For large linear gapes, the jaw was also gently translated anteriorly. Linear gapes smaller than 15 mm did not register measurable bite forces using this setup and were not included in the analyses. While sedated, each animal was weighed on a digital scale, and a series morphological measurements was recorded on the face using sliding calipers: jaw length (the distance between condylion laterale to infradentale), facial width (the distance between the left and right lateral canthus of the eyes), maximum gape (measured between the central incisors) and, if present, the amount of incisor overlap or spacing (following Hylander, 2013; Table 1).

**Table 1.** Morphological measurements for each *Macaca mulatta*

ID	Sex	Body mass (kg)	Eye width (mm)	Incisor overlap (mm)	Jaw length: condyle to I_1_ (mm)	Maximum gape at the incisors (mm)	Adjusted maximum gape (mm)
FZ	M	14.6	59.06	Tip to tip	115.73	63.04	63.04
HK	M	8.8	60.05	Open: 2.2	109.11	62.1	59.9
TM	M	14.2	59.12	Tip to tip	105.18	74.59	74.59
HD	F	6.2	54.77	Open: 1.85	87.5	46.3	44.45
HP	F	8.2	49.73	Tip to tip	89.92	44.14	44.14
LL	F	8.6	52.56	Open: 2.49	96.56	54.18	51.69
RT	F	7.1	51.63	Tip to tip	93.06	51.72	51.72
SR	F	5.4	50.17	Tip to tip	76.17	49.97	49.97

Incisor overlap scores and adjusted gape follow Hylander (2013).

Total bite force–gape data were collected for two of the macaques at the incisors (HK male, LL female) over a period of 8 months. A total of 419 voluntary bites (HK – 311; LL – 108) were collected from these animals between normal meal times, and neither animal was water or food restricted. Bites were recorded in 5 mm increments at linear gapes ranging from 10 to 55 mm for LL and between 15 and 60 mm for HK. Both animals received water-diluted apple or grape juice as a reward for biting, which was administered by a plastic cannula taped to the underside of the top bite plate. Bites were loosely threshold trained, meaning that the animal had to elicit stronger and stronger bites to receive a reward, and the reward was withheld for bites with lower force (described in Laird et al., 2023a). Only the maximum forces recorded at each gape for each animal were included in the analyses. Maximum linear gape at the central incisors measured under sedation was 63.76 mm for HK and 54.18 mm for LL. The largest linear gape for which active bites were recorded was 60 mm for HK and 55 mm for LL, suggesting that active force was measured at or near their maximum linear gapes. Active bite forces were isolated by subtracting the passive bite force data from the total bite force data.

### Behavior data

Bite force data in primates are best contextualized using multiple sources (Laird et al., 2025), and we supplemented the transducer forces for monkeys HK and LL using the shell fracture properties of two nuts. Both animals were provided with English walnuts and Brazil nuts in the shell (www.nuts.com) and filmed while feeding on these items using a Sony Handycam video camera (HD CRX405). Videos were reviewed to determine the location of fracture (incisors versus post-canine dentition). Force-to-fracture estimates for English walnut (233.39 N) and Brazil nut (614.46 N) shells were collected from the literature (Laird et al., 2023a,b). Dimensions of both nuts were collected using digital calipers (Tables S1 and S2).

### Passive and total bite force analyses

Maximum passive and total bite forces within each trial were segmented using the packages ‘pracma’ (https://CRAN.R-project.org/package=pracma), ‘quantmod’ (https://CRAN.R-project.org/package=quantmod) and ‘splitstackshape’ (https://CRAN.R-project.org/package=splitstackshape) in R (https://www.r-project.org/). We analyzed both linear and angular gape, which was calculated using jaw length measurements for each animal. Linear mixed effects models for linear and angular gape were used to test differences in passive tension between I_1_ (anterior) and M_1_ (posterior) locations on the toothrow and to test whether passive tension differed between males and females. Tests were conducted using the package ‘lme4’ (Bates et al., 2015) and *post hoc* tests in package ‘emmeans’ (https://CRAN.R-project.org/package=emmeans). Models for differences in passive tension at each bite point were constructed with the identification of each animal and sex as random factors, and sex was moved to an explanatory variable to test for differences between males and females. To determine whether forces at each linear gape were significantly greater than zero, separate models were fitted for anterior and posterior measurements with linear gape as a fixed effect and animal and sex as random effects. Estimated marginal means with 95% confidence intervals were used to identify linear gape levels where forces exceeded zero. These tests were only conducted on linear gape as a categorical variable. Significance level was set as 0.05.

### Construction of virtual bone and muscle segment models

#### 3D skeletal and muscle data

Muscle segment models were created using PLY mesh skull models downloaded from MorphoSource (Boyer et al., 2016) of an adult male and female *M. mulatta* (specimen IDs 000543170 and 000543165, respectively). These 3D meshes were cleaned and oriented such that the *y*-axis passes through the left and right TMJs and the *x*-axis is parallel to the premolar and molar occlusal plane using Geomagic Wrap 2017 (Geomagic, Morrisville, NC, USA). Full details on the muscle segments are described in Iriarte-Diaz et al. (2017) and Laird et al. (2024). Briefly, the three jaw adductor muscles were modeled using a series of seven equidistant segments aligned between the cranial and mandibular attachments and wrapped to the surface of the model. These 3D muscle models were used to simulate changes in gape by rotating the mandible from 0 to 50 deg at 5 deg increments. At each gape increment, the mandible was also translated so that the ramus cleared the postglenoid process and that the condyle remained in tangential contact with the glenoid fossa. For each gape, force position and direction were calculated for the virtual muscle segments. Muscle architecture data was obtained from Taylor and colleagues (2018) and is detailed in Table 2.

**Table 2.** *Macaca mulatta* muscle architecture data used in the models

	*L*_j_ (mm)	Superficial masseter			Temporalis		
		N*L*_f_ (mm)	PCSA (cm^2^)	α (deg)	N*L*_f_ (mm)	PCSA (cm^2^)	α (deg)
Female	83.59	13.08	5.11	15.42	16.52	11.76	13.02
Male	103.56	15.98	6.69	12.19	28.48	13.92	6.23

Data (means) are from Taylor et al. (2018). *L*_j_, jaw length; N*L*_f_, normalized fiber length; PCSA, physiological cross-sectional area; α, pennation angle.

The 3D model data (cranium, mandible and virtual muscles) and the muscle architecture data were scaled to match the jaw length of each subject using the measurements recorded from each animal during sedation. For linear variables such as fiber lengths, linear gapes, moment arms and muscle positions, values were scaled by multiplying the variable by *L*_j,exp_/*L*_j,MA_, where *L*_j,exp_ and *L*_j,MA_ are the jaw length of the specific experimental subject and the jaw length from the muscle architecture data, respectively. Physiological cross-sectional area (PCSA) was scaled by multiplying it by (*L*_j,exp_/*L*_j,MA_)^2^.

Linear gapes were transformed to angular gapes by measuring the linear distance between the upper and lower teeth (I_1_ for anterior bites and M_1_ for posterior bites) of the scaled 3D model at different gape angles, and then interpolating the experimental linear gapes.

To compare anterior and posterior bites, bite forces on posterior teeth (*F*_bite,M1_) were scaled to estimate bite forces on anterior teeth (*F*_bite,I1_) as:
(1)

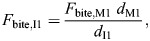
where *d*_I1_ and *d*_M1_ are the perpendicular distances of the first incisor and the first molar from the TMJ axis, respectively, where the TMJ axis is the line connecting the left and right TMJs.

#### Estimation of muscle and bite forces

Virtual 3D muscle models were created for one female and one male macaque. We created three different 3D models for each sex based on the active tension data collected from animals HK and LL: (1) a model without including passive tension, (2) a model similar to past approaches that optimizes both active and passive parameters to best fit the experimental bite force data, and (3) a model with individual-specific *in vivo* passive bite forces at the occlusal surface reported here.

Bite force at a particular bite point was estimated from the three jaw adductors, masseter, temporalis and medial pterygoid, by first estimating force for each muscle segment and then summing across the muscles.

Bite force produced by individual muscle segments for a given gape was estimated using the equation:
(2)

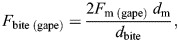
where *F*_m(gape)_ is the maximum force generated by an individual muscle segment at a given gape, *d*_m_ is the moment arm (perpendicular distance of the TMJ axis) of the segment of muscle and *d*_bite_ is the moment arm of the bite point and is multiplied by 2 to simulate the effect of both muscle sides.

*F*_m(gape)_ was calculated using a Hill-type model as:
(3)

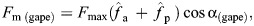
where 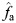 and 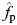 are the normalized active and passive force–length relationships (Fig. 1), α_(gape)_ is the pennation angle for a given gape, and *F*_max_ is the maximum tetanic force that the muscle segment can generate, calculated as:
(4)

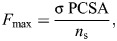
where σ represents the specific tension of skeletal muscle (estimated as ∼30 N cm^−2^ for 2M muscle fiber type), PCSA is the physiological cross-sectional area of the muscle (in cm^2^; see Laird et al., 2024; Taylor et al., 2025) and *n*_s_ indicates the number of segments per muscle.

The normalized active force–length curve was given by the equation (equation HH in Rockenfeller and Günther, 2017):
(5)

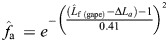
and the normalized passive force–length curve was defined by the equation (Dick et al., 2017):
(6)

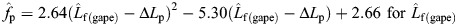

(7)

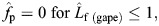
where 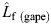 is the muscle fiber length for a given gape normalized to *L*_f,occ_, the fiber length at occlusion, and Δ*L*_a_ and Δ*L*_p_ are active and passive offset parameters, respectively, which are optimized for each subject (see ‘Optimization of muscle parameters’, below). Δ*L*_a_ represents how much a muscle fiber has to be stretched from occlusion to reach its optimal length, while Δ*L*_p_ represents the normalized slack length – how much the fiber has to be stretched to start generating passive force. 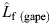 was calculated by the equation (Laird et al., 2024):
(8)

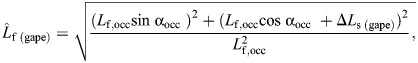
where α_occ_ is the pennation angle at occlusion and Δ*L*_s(gape)_ is the difference in the length of the muscle segment for a given gape with respect to the segment length at occlusion, measured directly from the 3D muscle models. Eqn 8 assumes that muscle fibers are parallel, coplanar and of equal width (Azizi and Deslauriers, 2014; Dick and Wakeling, 2018) and that as the length of the muscle changes, pennation angle varies while muscle thickness does not (see Laird et al., 2024).

#### Optimization of muscle parameters

Our muscle model requires the estimation of the active and passive offset parameters (Δ*L*_a_ and Δ*L*_p_) as well as of active and passive correction factors (cf_a_ and cf_p_), which affect the magnitude of the predicted bite force but do not change the shape of the bite force–gape relationship. All parameters were estimated using the MATLAB ‘lsqcurvefit’ function with the Levenberg–Marquardt algorithm, which searches for the best combination of parameters that minimizes the least-square differences between the predicted bite force values from the model and the *in vivo* data at different gapes.

For all individuals, we first estimated the passive parameters (Δ*L*_p_ and cf_p_) alone using the experimentally collected bite force data on anesthetized animals, which was used to determine the passive component of the bite force–gape relationship.

For the two individuals (HK and LL) with total bite force data collected experimentally, we tested three muscle models with different optimization conditions. The first model, ‘passive first’, uses the Δ*L*_p_ and cf_p_ passive parameters calculated above from the passive bite force data, and then only optimizes the active parameters (Δ*L*_a_ and cf_a_) that best fit the total bite force data. In this model, the cf_a_ and cf_p_ parameters can have different values. The second model, ‘total force’, optimized both the active parameters (Δ*L*_a_ and cf_a_) and the passive parameters (Δ*L*_p_ and cf_p_) together to best fit the experimental total bite force data only. In this model, the experimental passive bite force data are not used, and the cf_a_ and cf_p_ parameters are the same. The third model, ‘total force constrained’, optimizes both active and passive parameters to best fit the experimental total bite force data, but constrained so that the Δ*L*_a_ and Δ*L*_p_ parameters are the same, and the cf_a_ and cf_p_ parameters are the same.

## RESULTS

### Question 1: how do passive bite forces relate to gape and vary across the toothrow?

**Fig. 2.**
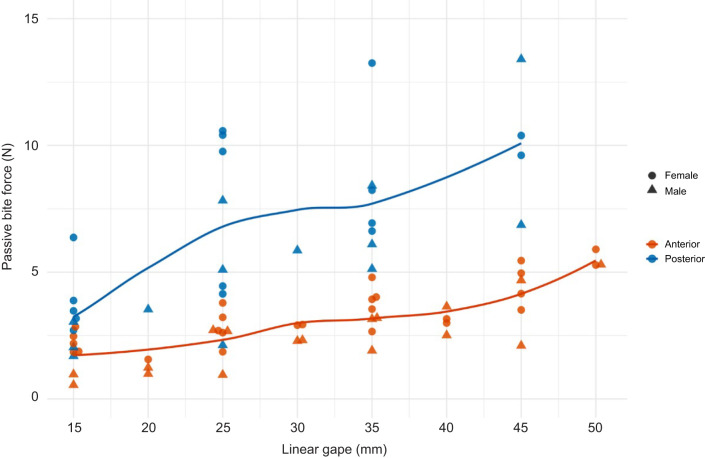
LOESS (locally estimated scatterplot smoothing) curves of passive bite forces across a range of linear gapes at anterior (I1) and posterior (M1) bite points for male and female *Macaca mulatta*.

Passive bite forces at both anterior and posterior bite points were significantly positively correlated with both linear and angular gape, such that higher passive bite forces were recorded at larger gapes (*P*<0.01; Fig. 2, Table 3; Table S3). Linear gapes below 15 mm did not register passive bite forces. As expected, passive bite forces differed between anterior and posterior bite points. A bite point at M_1_ was associated with passive bite forces that were 2.6 times larger than those on the anterior dentition (*P*<0.01). On the anterior dentition for linear gapes, females had significantly higher passive bite forces compared with males (*P*=0.03), but there were no significant differences between the sexes in passive bite forces at M_1_ for linear gape or for angular gape at the incisors or M_1_ (Table S3, Figs S1 and S2).

**Table 3.** *Macaca mulatta* passive bite force data

Location	Linear gape (mm)	Female		Male	
		*n*	Force (N) (range, mean)	*n*	Force (N) (range, mean)
Anterior	15	5	1.850–2.851, 2.249	2	0.550–0.971, 0.760
Anterior	20	1	1.561	3	0.887–1.234, 1.037
Anterior	25	5	1.862–3.790, 2.837	3	0.952–2.713, 2.113
Anterior	30	2	2.911–2.935, 2.923	3	2.042–2.321, 2.217
Anterior	35	5	2.660–4.795, 3.791	3	1.907–3.196, 2.752
Anterior	40	2	2.997–3.159, 3.078	2	2.512–3.645, 3.079
Anterior	45	4	3.514–5.455, 4.521	2	2.101–4.678, 3.389
Anterior	50	2	5.280–5.901, 5.590	1	5.298
Posterior	15	5	2.711–6.370, 3.923	3	1.692–3.044, 2.258
Posterior	20	–		1	3.534
Posterior	25	5	4.145–10.575, 7.868	3	2.124–7.826, 5.015
Posterior	30	–		1	5.85
Posterior	35	4	6.623–13.249, 8.761	3	5.128–8.415, 6.547
Posterior	40	–		–	
Posterior	45	2	9.608–10.389, 9.998	2	6.864–13.401, 10.132

*n* is the number of successful data points, which may include multiple data points from the same animal.

### Question 2: how do passive bite forces relate to peak active bite force and maximum gape?

Total bite forces were measured for one male (HK) and one female (LL) macaque. The maximum bite force for LL was 229.69 N measured at 25 mm linear gape, whereas the maximum for HK was 319.14 N recorded at 30 mm linear gape, which is the highest reported *in vivo* incisor force from a macaque (Table 4; Laird et al., 2025). Shelled nut fracture was used as a secondary measure for assessing bite force (Table S2). Macaque LL did not fracture English walnut or Brazil nut shells on the incisors, which is consistent with our transducer data. However, macaque HK fractured both the English walnut and Brazil nut shells using his incisors, which suggests that 319 N is an underestimate of his maximum total bite force.

**Table 4.** *Macaca mulatta* maximum total bite force data at each linear gape

Linear gape (mm)	LL		HK	
	No. of bites	Maximum bite force (N)	No. of bites	Maximum bite force (N)
10	1	26.03	0	NA
15	5	152.44	1	179.06
20	37	198.33	41	200.47
25	11	229.69	25	250.43
30	11	218.46	59	319.14
35	14	164.38	59	302.48
40	14	166.39	35	303.40
45	2	112.45	28	304.64
50	10	126.18	40	246.12
55	3	78.03	22	215.42
60	0	NA	1	168.91

Bite force data are for a female (LL) and male (HK) *M. mulatta*.

**Fig. 3.**
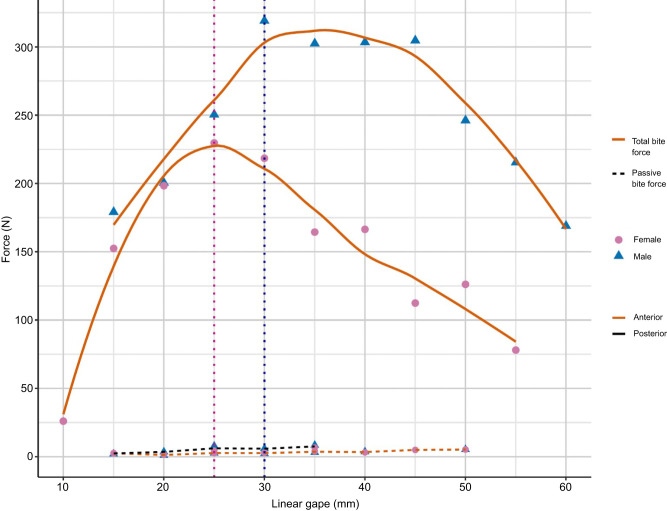
**Total and passive bite force data.** Maximum total forces (LOESS dashed lines) come from two animals (HK male and LL female) and the maximum passive forces (solid lines) reflect data from eight macaques. The two vertical dashed lines represent maximum total bite forces for LL (left; pink) and HK (right; blue).

As expected, total bite forces in both animals increased with larger gapes until reaching their respective maxima at 25 mm (LL) and 30 mm (HK) linear gape, after which bite force decreased with larger gapes (Fig. 3; Fig. S2). Across all animals, confidence intervals for the relationship between anterior passive bite forces and gape indicate that forces at linear gapes larger than 30 mm were significantly different from zero, and posterior passive bite forces were significantly different from zero for linear gapes larger than 25 mm (Table S4). Differences in confidence intervals for males and females could not be tested because of the low sample sizes. This suggests passive bite forces significantly differ from zero around maximum gape; however, total forces in both animals were substantially larger than passive bite forces at all gapes, including at maximum gape.

### Question 3: how do passive muscle forces influence Hill-type muscle model predictions of bite force and operating range?

**Fig. 4.**
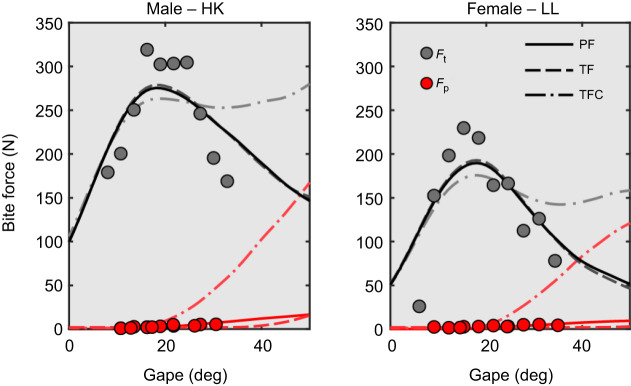
**Comparison of experimentally collected data on male and female *M. mulatta* with predictions from different muscle models.** Gray circles are total bite force data (*F*_t_) collected on HK and LL while awake and red circles are bite forces collected on anesthetized animals, representing passive bite forces (*F*_p_). Three different models were fitted to the experimental data: (1) ‘passive first’ (PF), where passive force parameters were optimized first using experimental passive bite force data, and then these parameters were used to optimize the active force parameters; (2) ‘total force’ (TF), where passive and active force parameters were optimized together using the total bite force data only; and (3) ‘total force constrained’ (TFC), where passive and active force components were optimized together with total bite force data only, but Δ*L*_a_ and Δ*L*_p_ were constrained to have the same value.

Optimized passive parameters based on the passive bite force data show that the Δ*L*_p_ ranged from 0.25 to 0.43, but the correction factor cf_p_ indicates that passive bite forces were largely overestimated by the model (cf_p_<0.1; Table S5, Fig. S2). For the two individuals with total bite force data, two of the muscle models fitted the experimental data reasonably well (*R*^2^>0.58; models ‘passive first’ and ‘total force’) while one model overestimated bite forces at large gapes (*R*^2^<0.46; ‘total force constrained’; Fig. 4). Models ‘passive first’ and ‘total force’ had similar active offset parameters Δ*L*_a_ between 0.47 and 0.52, but they differed in their passive offset parameters (Table 5). Model ‘passive first’ had Δ*L*_p_ values between 0.25 and 0.33, indicating that the passive muscle forces start developing before the muscle fibers reach their optimal length (Δ*L*_p_<Δ*L*_a_), but increase very slowly with gape. In contrast, the ‘total force’ model had Δ*L*_p_ values well above the optimal muscle length (Δ*L*_p_>Δ*L*_a_). This resulted in a model that effectively does not have a passive bite force component within the range of gapes measured. In other words, this model represents a model with only an active force component (Fig. 4). Finally, the ‘total force constrained’ model also had similar Δ*L*_a_ values to the ‘passive first’ and the ‘total force’ models, but the Δ*L*_p_ was constrained to be the same as Δ*L*_a_. In this model, the passive muscle force component started increasing substantially at around 20 deg gapes, overestimating the measured passive bite force values.

**Table 5.** Optimized passive and active parameters for the three modeled muscle conditions

ID	Sex	Passive first (PF)					Total force (TF)					Total force constrained (TFC)				
		Passive		Active		*R* ^2^	Passive		Active		*R* ^2^	Passive		Active		*R* ^2^
		Δ*L*_p_	cf_p_	Δ*L*_a_	cf_a_		Δ*L*_p_	cf_p_	Δ*L*_a_	cf_a_		Δ*L*_p_	cf_p_	Δ*L*_a_	cf_a_	
HK	M	0.24	0.09	0.47	1.60	0.58	1.17	1.63	0.48	1.63	0.61	0.44	1.47	0.44	1.47	0.31
LL	F	0.31	0.06	0.51	1.27	0.64	1.38	1.29	0.52	1.29	0.64	0.50	1.16	0.50	1.16	0.46

Δ*L*_p_/Δ*L*_a_, change in passive/active offset parameter; cf_p_/cf_a_, passive/active correction factor.

## DISCUSSION

Measures of passive muscle force of the mammalian feeding system are limited (but see Thexton and Hiiemae, 1975; Anapol and Herring, 1989; Konow et al., 2025), although the expectation is that passive muscle forces are on par with, or exceed, active force when stretched based on a Hill-type length–tension model (Fig. 1; Hill, 1938, 1950). Here, we tested how *in vivo* passive and total bite forces vary with gape in a primate, the rhesus macaque, and found that passive bite force is substantially lower than active force, even at large gapes. We acknowledge that our total force sample is limited to two animals, and that the use of sedatives has previously been noted to increase muscle stiffness (e.g. tongue stiffness under general anesthesia, Kappert et al., 2021), potentially affecting the jaw adductors. However, increased muscle stiffness would have limited gape stretch, resulting in higher passive bite force values than what might be expected under other sedation methods. Our passive bite force values may therefore be an upper bound, and are still substantially lower than total bite force values. Further, sedatives, including midazolam and dexmedetomidine, have been linked with increased bite force production through the loss of the proprioceptive function of the periodontal ligament (Sivasubramani et al., 2019), effectively suppressing the body's natural neural feedback loop for risking damage to the system. This study is the first to compare primate *in vivo* passive and active bite forces at the occlusal surface, and we discuss the implications of these data for bite force–gape tradeoffs and muscle models of the feeding system.

To contextualize the total bite force data, we compared our data with the extensive literature on bite forces in macaques (reviewed in Laird et al., 2025). We report the highest *in vivo* bite forces for adult male and female macaques measured using a force transducer on the incisors (male: 319.14 N; female: 229.69 N). These values are more than twice the magnitude of previously reported *in vivo* and stimulated incisor forces (Hylander, 1979; Dechow and Carlson, 1990), but smaller than reported incisor force values estimated using food fracture, muscle architecture and jaw leverage (Hill et al., 1995; Deutsch et al., 2020; Holmes and Taylor, 2023). These data are also consistent with our behavior data, although 319.14 N may be an underestimate of animal HK's incisor bite force. We also compared our maximum gape values with previous studies. All but one of the animals measured here had maximum gapes smaller than the reported male and female ranges of a sample of 50 *M. mulatta* (Hylander, 2013). However, all maximum gapes for the animals in this study were within their respective male and female ranges for a sample of 117 adult rhesus macaques from Cayo Santiago, Puerto Rico (Canington et al., 2026).

### Primate jaw adductor passive forces are small across the jaw's operational range

Collectively, our results demonstrate that *M. mulatta* passive bite forces across the toothrow increase with gape. This means that as the jaw opens, the jaw adductor muscles are stretched, resulting in the generation of tensile forces from the connective tissue, proteins (e.g. titin) and other structural elements in the absence of active contraction (Gordon et al., 1966; Lazarides, 1980; Labeit and Kolmerer, 1995; Purslow, 2020). As expected, passive bite forces were larger at M_1_ than at the incisors for comparable linear and angular gapes. This difference reflects the shorter moment arm of an M_1_ bite point to the TMJ, and the larger amount of gape needed to open the jaw to a particular angle or linear distance at a posterior bite point compared with a bite point at the incisors (Greaves, 1978). Our results also suggest a few differences in passive bite forces between males and females, as the only measure demonstrating significant sexual dimorphism was passive bite force at the incisors across linear gapes (Table S3). Muscle architecture data from *M. fascicularis* suggest males have longer jaw adductor fibers compared with females (Terhune et al., 2015), and coupled with males having larger maximum gapes (Table 1; Hylander, 2013), this implies that passive bite forces generated by the jaw adductors in males should be lower than those in females for a given gape. While this pattern held for our incisive measures across linear gapes, our total sample size was small. We expect greater sexual dimorphism in passive bite forces with increased sampling.

**Fig. 5.**
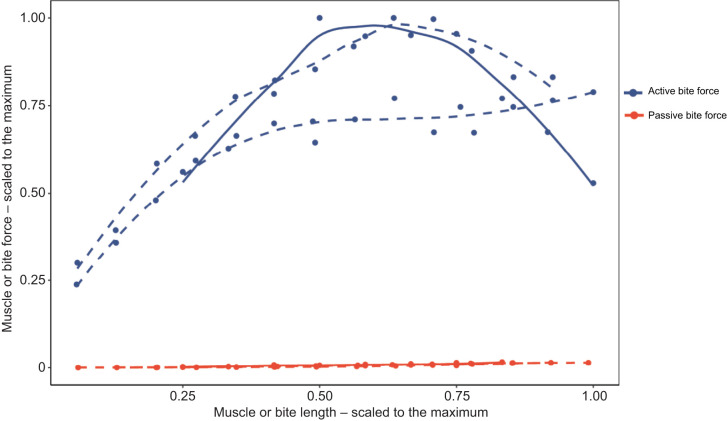
**Active and passive bite forces at the occlusal surface in *M. mulatta* and active and passive temporalis muscle forces in *Didelphis virginiana*.** The passive data for the Virgina opossum, *D. virginiana*, from Thexton and Hiiemae (1975) were originally scaled by 100. After unscaling these passive data, both the active and passive force data from both taxa were scaled to their respective active maxima, making them directly comparable. Solid lines represent data from this study; dashed lines represent data from Thexton and Hiiemae (1975).

Previous studies of passive tension in the feeding system noted that passive forces began to increase prior to peak active force (Fig. 1; Thexton and Hiiemae, 1975; Anapol and Herring, 1989; Konow et al., 2025). Our data were consistent with these prior findings. We found that passive bite forces significantly differed from zero around peak total bite force in our two animals (Figs 3 and 5). Our data are also in line with previously published opossum data (Thexton and Hiiemae, 1975), with passive bite forces neither exceeding nor nearing active bite forces, despite recording close to maximum gape. However, the macaque passive forces differed from those of minipigs (Anapol and Herring, 1989), and to a lesser degree rats (Konow et al., 2025), which were more similar to the Hill-type model expectations. The similarity between macaques and opossums is notable as both taxa use large gapes associated with different behaviors. *Didelphis virginiana* is known to hold large gapes for display as part of their fear responses, but this behavior is rarely accompanied by an attack (McManus, 1970). Holding a wide gape without generating large active bite forces may seemingly benefit from high passive tension, as predicted by a Hill model, which could theoretically stabilize the TMJ, allowing gape to be maintained with low adductor muscle activity. These results are also consistent with modeled passive tension data in gouging marmosets and non-gouging tamarins, as larger modeled passive tension values were reported in non-gouging tamarins (Eng et al., 2009).

Macaques accompany large gapes with aggressive and defensive behaviors involving active bite force generation, such as canine biting (Altmann, 1967; Plavcan and van Schaik, 1992; Hylander, 2013; Terhune et al., 2015). Despite this difference in the use of force at large gapes, both macaques and opossums had similar passive estimates, indicating that passive forces play a minimal role in constraining the TMJ and are minimally influenced by large gape behaviors. Anatomical differences between these taxa (e.g. stiffness of connective tissues and mechanical advantage) may help us elucidate how passive costs are similarly achieved despite behavioral differences.

Despite similarities in the findings, there are notable differences in approach between our study and those referenced here. Past studies tested single muscles and estimated active forces using nerve cuff stimulations, and nerve cuffs are unlikely to generate maximum active tension across the entire muscle (Anapol and Herring, 1989). We analyzed both passive and total bite forces at the teeth, which means our estimates included contributions from all of the jaw adductor muscles in their anatomical positions. Functionally heterogeneous muscles, such as the anterior and posterior regions of temporalis, were therefore able to undergo differing amounts of stretch that may have contributed to our low passive muscle forces (Laird et al., 2020, 2023a,b). Passive muscle forces also differ depending on connective tissue and individual muscle structure (Lieber et al., 2025). Thus, measuring passive and total bite forces at the teeth, as opposed to isolated muscles, is inclusive of all jaw adductors within the geometry of the feeding system, which can inform the biological context for these forces. However, additional tests of passive bite force generation are needed across the jaw adductors and between single muscles and the teeth.

Within a single muscle, passive forces provide limits on muscle stretch to avoid structural damage. Passive bite forces are similarly proposed to limit gape and minimize distractive forces at the TMJ in the feeding system, minimizing the risk of structural damage (as seen in the locomotor system; Horner et al., 2024). However, we found that primates do not reach large passive bite forces, even near maximum gape. This suggests that passive forces provide few constraints and protective mechanisms at the TMJ during large gape behaviors in macaques. The bony morphology of the TMJ may instead prevent the jaw adductor muscles from reaching high levels of passive tension (Terhune et al., 2022). However, we caution that this may not be true in all primates. Strepsirrhines have a broad, shallow TMJ with fewer bony constraints compared with the TMJ of an anthropoid primate (Terhune et al., 2022). However, a recent re-evaluation of the constrained lever model suggests that no primates have distractive forces at the TMJ at gapes larger than 15 deg (Iriarte-Diaz et al., 2026).

### Passive forces play a minor role in Hill-type muscle models of the feeding system

The estimated parameters of the Hill-type muscle models, where passive and active force components were optimized independently (‘passive first’), show that passive forces start increasing on the ascending limb of the length–tension curve, approximately at 0.8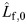, as has been observed for jaw adductor muscles in other mammals (Thexton and Hiiemae, 1975; Anapol and Herring, 1989). However, our data show that passive muscle forces increase very slowly with gape. Our *in vivo* passive bite forces are much lower than predicted by the muscle models (11 times lower for HK and 16 times lower for LL). A possible explanation for such overestimation could be that the model input parameters overestimate the size of the experimental animals. However, this explanation seems unlikely, considering that the same model underestimated the total bite force in both experimental subjects (by 60% and 27% for HK and LL, respectively). The ‘total force’ models were fitted using only total bite force data, and were similar to the ‘passive first’ models (Fig. 5), further suggesting that passive components have little impact on the generation of force in the jaw adductor muscles of the rhesus macaque.

These results are drastically different from the bite forces predicted by traditional Hill-type models (‘total force constrained’; Fig. 5), where passive muscle forces are only present beyond the optimal muscle length, increasing exponentially so that at 50% muscle stretch, passive tension equals maximum active force (Otten, 1987; Zajac, 1989). This is an unexpected result, considering that passive forces have been shown to be larger in whole muscles than in single muscle fibers or bundles of fibers as a result of the greater importance of the extracellular matrix (Ward et al., 2020). To be able to compare our data with other muscles and organisms, we calculated the *L*_20_, the relative muscle length that develops 20% of the maximal relative muscle force, for the jaw adductor muscles evaluated here, which ranged between 1.7 and 1.8. This is substantially higher than the range (*L*_20_=1.1) observed for hindlimb muscles in mammals (between 1 and 1.44; Azizi and Roberts, 2010; Horner et al., 2024), and even higher than the average for anurans (*L*_20_=1.5; Azizi and Roberts, 2010). This high muscle compliance observed in anurans is thought to be necessary for the large joint excursions associated with jumping, as it has been suggested that increased passive muscle stiffness can limit the muscle's range of motion (Brown et al., 1996; Horner et al., 2024). The only data on jaw muscles, to our knowledge, come from the jaw adductor muscles of anole lizards, with *L*_20_ ranging from 1.24 to 1.37 (Anderson and Roberts, 2020). Together, these findings demonstrate that macaque jaw adductor muscles exhibit unusually high compliance relative to both limb and jaw muscles of other animals that may relate to selection for large gape behaviors. These data highlight a departure from existing muscle models and present a new framework for comparative analyses of musculoskeletal design.

## Supplementary Material

10.1242/jexbio.251950_sup1 Supplementary information

## References

[JEB251950C1] Aguirre, L. F., Herrel, A., Van Damme, R. and Matthysen, E. (2003). The implications of food hardness for diet in bats. *Funct. Ecol.* 17, 201-212. 10.1046/j.1365-2435.2003.00721.x

[JEB251950C2] Altmann, S. A. (1967). The structure of primate social communication. In *Social Communication Among Primates* (ed. S.A. Altmann), pp. 3325-3362. Chicago: University of Chicago Press.

[JEB251950C3] Anapol, F. and Herring, S. W. (1989). Length-tension relationships of masseter and digastric muscles of miniature swine during ontogeny. *J. Exp. Biol.* 143, 1-16. 10.1242/jeb.143.1.1 2732657

[JEB251950C4] Anderson, F. C. and Pandy, M. G. (2003). Individual muscle contributions to support in normal walking. *Gait Posture* 17, 159-169. 10.1016/S0966-6362(02)00073-5 12633777

[JEB251950C5] Anderson, C. V. and Roberts, T. J. (2020). The need for speed: functional specializations of locomotor and feeding muscles in *Anolis* lizards. *J. Exp. Biol.* 223, jeb213397. 10.1242/jeb.213397 31862851

[JEB251950C6] Azizi, E. and Deslauriers, A. R. (2014). Regional heterogeneity in muscle fiber strain: the role of fiber architecture. *Front. Physiol.* 5, 103613. 10.3389/fphys.2014.00303 PMC4129366 25161626

[JEB251950C7] Azizi, E. and Roberts, T. J. (2010). Muscle performance during frog jumping: influence of elasticity on muscle operating lengths. *Proc. R. Soc. B* 277, 1523-1530. 10.1098/rspb.2009.2051 PMC2871832 20106852

[JEB251950C8] Bates, D., Mächler, M., Bolker, B. and Walker, S. (2015). Fitting linear mixed-effects models using lme4. *J. Stat. Softw.* 67, 1-48. 10.18637/jss.v067.i01

[JEB251950C9] Biewener, A. A. (1998). Muscle-tendon stresses and elastic energy storage during locomotion in the horse. *Comp. Biochem. Physiol. B Biochem. Mol. Biol.* 120, 73-87. 10.1016/S0305-0491(98)00024-8 9787779

[JEB251950C10] Biewener, A. A., Wakeling, J. M., Lee, S. S. and Arnold, A. S. (2014). Validation of Hill-type muscle models in relation to neuromuscular recruitment and force–velocity properties: predicting patterns of in vivo muscle force. *Integr. Comp. Biol.* 54, 1072-1083. 10.1093/icb/icu070 24928073 PMC4296201

[JEB251950C11] Boyer, D. M., Gunnell, G. F., Kaufman, S. and McGeary, T. M. (2016). Morphosource: archiving and sharing 3-D digital specimen data. *Paleontol. Soc. Pap.* 22, 157-181. 10.1017/scs.2017.13

[JEB251950C12] Brown, I. E., Cheng, E. J. and Loeb, G. E. (1996). Measured and modeled properties of mammalian skeletal muscle. II. The effects of stimulus frequency on force–length and force–velocity relationships. *J. Muscle Res. Cell. Motil.* 20, 627-643. 10.1023/A:1005585030764 10672511

[JEB251950C75] Canington, S., Turcotte, C., Hernandez-Janer, E., Stock, M., Villamil, C. I., Montague, M., Martinez, M., Williams, S., Antón, S., Higham, J. and Laird, M. (2026). Maximum gape and jaw leverage in developing rhesus macaques (*Macaca mulatta*). Program of the 95th Annual Meeting of the American Association of Biological Anthropologists. *Am. J. Biol. Anthropol.* 189, Suppl. 81, e70227. 10.1002/ajpa.70227 41823377

[JEB251950C14] Corlett, R. T. and Lucas, P. W. (1990). Alternative seed-handling strategies in primates: seed-spitting by long-tailed macaques (*Macaca fascicularis*). *Oecologia* 82, 166-171. 10.1007/BF00323531 28312661

[JEB251950C15] Davis, J., Kaufman, K. R. and Lieber, R. L. (2003). Correlation between active and passive isometric force and intramuscular pressure in the isolated rabbit tibialis anterior muscle. *J. Biomech.* 36, 505-512. 10.1016/S0021-9290(02)00430-X 12600341

[JEB251950C16] Dechow, P. C. and Carlson, D. S. (1990). Occlusal force and craniofacial biomechanics during growth in rhesus monkeys. *Am. J. Phys. Anthropol.* 83, 219-237. 10.1002/ajpa.1330830211 2248381

[JEB251950C17] Delp, S. L., Anderson, F. C., Arnold, A. S., Loan, P., Habib, A., John, C. T., Guendelman, E. and Thelen, D. G. (2007). OpenSim: Open-source software to create and analyze dynamic simulations of movement. *IEEE Trans. Biomed. Eng.* 54, 1940-1950. 10.1109/TBME.2007.901024 18018689

[JEB251950C18] Deutsch, A. R., Dickinson, E., Leonard, K. C., Pastor, F., Muchlinski, M. N. and Hartstone-Rose, A. (2020). Scaling of anatomically derived maximal bite force in primates. *Anat. Rec.* 303, 2026-2035. 10.1002/ar.24284 31587507

[JEB251950C19] Dick, T. J. M. and Wakeling, J. M. (2018). Geometric models to explore mechanisms of dynamic shape change in skeletal muscle. *R. Soc. Open Sci.* 5, 172371. 10.1098/rsos.172371 29892420 PMC5990834

[JEB251950C20] Dick, T. J. M., Biewener, A. A. and Wakeling, J. M. (2017). Comparison of human gastrocnemius forces predicted by Hill-type muscle models and estimated from ultrasound images. *J. Exp. Biol.* 220, 1643-1653. 10.1242/jeb.154807 28202584 PMC5450802

[JEB251950C21] Eng, C. M., Ward, S. R., Vinyard, C. J. and Taylor, A. B. (2009). The morphology of the masticatory apparatus facilitates muscle force production at wide jaw gapes in tree-gouging common marmosets (*Callithrix jacchus*). *J. Exp. Biol.* 212, 4040-4055. 10.1242/jeb.029983 19946083 PMC4075048

[JEB251950C22] Ettema, G. J. C. (1996). Mechanical efficiency and efficiency of storage and release of series elastic energy in skeletal muscle during stretch–shorten cycles. *J. Exp. Biol.* 199, 1983-1997. 10.1242/jeb.199.9.1983 8831144

[JEB251950C23] Gordon, A. M., Huxley, A. F. and Julian, F. J. (1966). The variation in isometric tension with sarcomere length in vertebrate muscle fibres. *J. Physiol. (Lond)* 184, 170-192. 10.1113/jphysiol.1966.sp007909 5921536 PMC1357553

[JEB251950C24] Greaves, W. S. (1978). The jaw lever system in ungulates: a new model. *J. Zool.* 184, 271-285. 10.1111/j.1469-7998.1978.tb03282.x

[JEB251950C25] Horner, A. M., Azizi, E. and Roberts, T. J. (2024). The interaction of in vivo muscle operating lengths and passive stiffness in rat hindlimbs. *J. Exp. Biol.* 227, jeb246280. 10.1242/jeb.246280 38353270 PMC10984277

[JEB251950C26] Thelen, D. G. (2003). Adjustment of muscle mechanics model parameters to simulate dynamic contractions in older adults. *J. Biomech. Eng.* 125, 70-77. 10.1115/1.1531112 12661198

[JEB251950C27] Herrel, A., R. Van Damme, B. Vanhooydonck, and F. and De Vree, (2001). The implications of bite performance to diet in two species of lacertid lizards. *Can. J. Zool.* 79: 662-670. 10.1139/z01-031

[JEB251950C28] Herrel, A., Vanhooydonck, B. and Van Damme, R. (2004). Omnivory in lacertid lizards: adaptive evolution or constraint? *J. Evol. Biol.* 17, 974-984. 10.1111/j.1420-9101.2004.00758.x 15312070

[JEB251950C29] Herrel, A., Podos, J., Huber, S. K. and Hendry, A. P. (2005). Evolution of bite force in Darwin's finches: a key role for head width. *J. Evol. Biol.* 18, 669-675. 10.1111/j.1420-9101.2004.00857.x 15842496

[JEB251950C30] Hill, A. V. (1938). The heat of shortening and the dynamic constants of muscle. *Proc. R. Soc. Lond. B Biol. Sci.* 126, 136-195. 10.1098/rspb.1938.0050 18152150

[JEB251950C31] Hill, A. V. (1950). The dimensions of animals and their muscular dynamics. *Sci. Prog.* 38, 209-230.

[JEB251950C32] Hill, D. A., Lucas, P. W. and Cheng, P. Y. (1995). Bite forces used by Japanese macaques (*Macaca fuscata yakui*) on Yakushima Island, Japan to open aphid-induced galls on *Distylium racemosum* (Hamamelidaceae). *J. Zool.* 237, 57-63. 10.1111/j.1469-7998.1995.tb02746.x

[JEB251950C33] Holmes, M. and Taylor, A. B. (2023). Modeling mechanical advantage from fossils: What are we missing? *Am. J. Biol. Anthropol.* 180, 77. 10.1002/ajpa.24639

[JEB251950C34] Hylander, W. L. (1979). The functional significance of primate mandibular form. *J. Morphol.* 160, 223-239. 10.1002/jmor.1051600208 458862

[JEB251950C35] Hylander, W. L. (2013). Functional links between canine height and jaw gape in catarrhines with special reference to early hominins. *Am. J. Phys. Anthropol.* 150, 247-259. 10.1002/ajpa.22195 23280236

[JEB251950C36] Iriarte-Diaz, J., Terhune, C. E., Taylor, A. B. and Ross, C. F. (2017). Functional correlates of the position of the axis of rotation of the mandible during chewing in non-human primates. *Zoology* 124, 106-118. 10.1016/j.zool.2017.08.006 28993018

[JEB251950C37] Iriarte-Diaz, J., Martin, A. and Laird, M. F. (2026). A reevaluation of the constrained lever model in the primate feeding system. *J. Exp. Biol.* 229, jeb251335. 10.1242/jeb.251335 41808540

[JEB251950C38] Kappert, K. D. R., Connesson, N., Elahi, S. A., Boonstra, S., Balm, A. J. M., van Der Heijden, F. and Payan, Y. (2021). *In-vivo* tongue stiffness measured by aspiration: resting vs general anesthesia. *J. Biomech.* 114, 110147. 10.1016/j.jbiomech.2020.110147 33276256

[JEB251950C39] Konow, N., Reder, B., Bartlett, D., Jenness, D., Patel, T., Moore, J. R. and Brocklehurst, R. J. (2025). Rat superficial masseter operates at long lengths during biting. *Sci. Rep.* 15, 37978. 10.1038/s41598-025-21953-z 41168355 PMC12575779

[JEB251950C40] Labeit, S. and Kolmerer, B. (1995). Titins: giant proteins in charge of muscle ultrastructure and elasticity. *Science* 270, 293-296. 10.1126/science.270.5234.293 7569978

[JEB251950C41] Laird, M. F., Vogel, E. R. and Pontzer, H. (2016). Chewing efficiency and occlusal functional morphology in modern humans. *J. Hum. Evol.* 93, 1-11. 10.1016/j.jhevol.2015.11.005 27086052

[JEB251950C42] Laird, M. F., Granatosky, M. C., Taylor, A. B. and Ross, C. F. (2020). Muscle architecture dynamics modulate performance of the superficial anterior temporalis muscle during chewing in capuchins. *Sci. Rep.* 10, 6410. 10.1038/s41598-020-63376-y 32286442 PMC7156371

[JEB251950C43] Laird, M. F., Kanno, C. M., Yoakum, C. B., Fogaça, M. D., Taylor, A. B., Ross, C. F., Chalk-Wilayto, J., Holmes, M. A., Terhune, C. E. and de Oliveira, J. A. (2023a). Ontogenetic changes in bite force and gape in tufted capuchins. *J. Exp. Biol.* 226, jeb245972. 10.1242/jeb.245972 37439316

[JEB251950C44] Laird, M. F., Iriarte-Diaz, J., Byron, C. D., Granatosky, M. C., Taylor, A. B. and Ross, C. F. (2023b). Gape drives regional variation in temporalis architectural dynamics in tufted capuchins. *Philos. Trans. R. Soc. B* 378, 20220550. 10.1098/rstb.2022.0550 PMC10577035 37839440

[JEB251950C45] Laird, M. F., Polvadore, T. A., Hirschkorn, G. A., McKinney, J. C., Ross, C. F., Taylor, A. B., Terhune, C. E. and Iriarte-Diaz, J. (2024). Tradeoffs between bite force and gape in *Eulemur* and *Varecia*. *J. Morphol.* 285, e21699. 10.1002/jmor.21699 38715161

[JEB251950C46] Laird, M. F., Holmes, M. A., Terhune, C. E. and Taylor, A. B. (2025). A (Bite) force to be reckoned with. *Am. J. Biol. Anthropol.* 188, e70144. 10.1002/ajpa.70144 41152196 PMC12568753

[JEB251950C47] Langenbach, G. E. J. and Hannam, A. G. (1999). The role of passive muscle tensions in a three-dimensional dynamic model of the human jaw. *Arch. Oral Biol.* 44, 557-573. 10.1016/S0003-9969(99)00034-5 10414871

[JEB251950C48] Lazarides, E. (1980). Intermediate filaments as mechanical integrators of cellular space. *Nature (Lond.)* 283, 249-256. 10.1038/283249a0 7188712

[JEB251950C49] Lieber, R. L., Wang, Z., Binder-Markey, B. I., Persad, L. S., Shin, A. Y. and Kaufman, K. R. (2025). Modeling implications of the relationship between active and passive skeletal muscle mechanical properties. *J. Biomech.* 178, 112423. 10.1016/j.jbiomech.2024.112423 39631228 PMC12912294

[JEB251950C50] McManus, J. J. (1970). Behavior of captive opossums, *Didelphis marsupialis virginiana*. *Am. Midl. Nat.* 84, 144-169. 10.2307/2423733

[JEB251950C51] Miller, A. J. (1991). *Craniomandibular Muscles: their Role in Function and Form*. CRC Press.

[JEB251950C52] Otten, E. (1987). A myocybernetic model of the jaw system of the rat. *J. Neurosci. Methods* 21, 287-302. 10.1016/0165-0270(87)90123-3 3682879

[JEB251950C53] Panagiotopoulou, O., Robinson, D., Iriarte-Diaz, J., Ackland, D., Taylor, A. B. and Ross, C. F. (2023). Dynamic finite element modelling of the macaque mandible during a complete mastication gape cycle. *Philos. Trans. R. Soc. B* 378, 20220549. 10.1098/rstb.2022.0549 PMC10577025 37839457

[JEB251950C54] Peterson, M. D., Pistilli, E., Haff, G. G., Hoffman, E. P. and Gordon, P. M. (2011). Progression of volume load and muscular adaptation during resistance exercise. *Eur. J. Appl. Physiol.* 111, 1063-1071. 10.1007/s00421-010-1735-9 21113614 PMC4215195

[JEB251950C55] Plavcan, J. M. and van Schaik, C. P. (1992). Intrasexual competition and canine dimorphism in anthropoid primates. *Am. J. Phys. Anthropol.* 87, 461-477. 10.1002/ajpa.1330870407 1580353

[JEB251950C56] Purslow, P. P. (2020). The structure and role of intramuscular connective tissue in muscle function. *Front. Physiol.* 11, 495. 10.3389/fphys.2020.00495 32508678 PMC7248366

[JEB251950C57] Rockenfeller, R. and Günther, M. (2017). How to model a muscle's active force–length relation: a comparative study. *Comput. Methods Appl. Mech. Eng.* 313, 321-336. 10.1016/j.cma.2016.10.003

[JEB251950C58] Ross, and Iriarte-Diaz, (2019). Evolution, constraint, and optimality in primate feeding systems. In *Feeding in Vertebrates: Evolution, Morphology, Behavior, Biomechanics* (ed. V. Bels), pp. 787-829. Cham: Springer International Publishing.

[JEB251950C59] Rugh, J. D., Drago, C. J. and Barghi, N. (1980). Comparison of electromyographic and phonetic measurements of vertical rest position. *J. Prosthet. Dent.* 44, 450. 10.1016/0022-3913(80)90108-0

[JEB251950C60] Sivasubramani, S. M., Pandyan, D. A., Chinnasamy, R. and Kumar Kuppusamy, S. (2019). Comparison of bite force after administration of midazolam and dexmedetomidine for conscious sedation in minor oral surgery. *J. Pharm. Bioallied Sci.* 11 Suppl. 2, S446-S449. 10.4103/JPBS.JPBS_67_19 31198385 PMC6555307

[JEB251950C61] Taylor, A. B., Terhune, C. E., Toler, M., Holmes, M., Ross, C. F. and Vinyard, C. J. (2018). Jaw-muscle fiber architecture and leverage in the hard-object feeding sooty mangabey are not structured to facilitate relatively large bite forces compared to other papionins. *Anat. Rec.* 301, 325-342. 10.1002/ar.23718 29330952

[JEB251950C62] Taylor, A. B., Terhune, C. E., Ross, C. F. and Vinyard, C. J. (2024). Jaw-muscle fiber architecture and skull form facilitate relatively wide jaw gapes in male cercopithecoid monkeys. *J. Hum. Evol.* 197, 103601. 10.1016/j.jhevol.2024.103601 39500178

[JEB251950C63] Taylor, A. B., Holmes, M. A., Laird, M. F. and Terhune, C. E. (2025). Jaw-muscle structure and function in primates: insights into muscle performance and feeding-system behaviors. *Evol. Anthropol.* 34, e22053. 10.1002/evan.22053 39964129 PMC11834762

[JEB251950C64] Terhune, C. E., Hylander, W. L., Vinyard, C. J. and Taylor, A. B. (2015). Jaw-muscle architecture and mandibular morphology influence relative maximum jaw gapes in the sexually dimorphic Macaca fascicularis. *J. Hum. Evol.* 82, 145-158. 10.1016/j.jhevol.2015.02.006 25858337

[JEB251950C65] Terhune, C. E., Mitchell, D. R., Cooke, S. B., Kirchhoff, C. A. and Massey, J. S. (2022). Temporomandibular joint shape in anthropoid primates varies widely and is patterned by size and phylogeny. *Anat. Rec.* 305, 2227-2248. 10.1002/ar.24886 35133075

[JEB251950C66] Thexton, A. J. and Hiiemae, K. M. (1975). The twitch-contraction characteristics of opossum jaw musculature. *Arch. Oral Biol.* 20, 743-748. 10.1016/0003-9969(75)90046-1 1061528

[JEB251950C67] Van Casteren, A., Codd, J. R., Kupczik, K., Plasqui, G., Sellers, W. I. and Henry, A. G. (2022). The cost of chewing: the energetics and evolutionary significance of mastication in humans. *Sci. Adv.* 8, eabn8351. 10.1126/sciadv.abn8351 35977013 PMC9385136

[JEB251950C68] van Eijden, T. M. G. J., Turkawski, S. J. J., van Ruijven, L. J. and Brugman, P. (2002). Passive force characteristics of an architecturally complex muscle. *J. Biomech.* 35, 1183-1189. 10.1016/S0021-9290(02)00087-8 12163308

[JEB251950C69] Verwaijen, D., Van Damme, R. and Herrel, A. (2002). Relationships between head size, bite force, prey handling efficiency and diet in two sympatric lacertid lizards. *Funct. Ecol.* 16, 842-850. 10.1046/j.1365-2435.2002.00696.x

[JEB251950C70] Wall, C. E., Hanna, J. B., O'Neill, M. C., Toler, M. and Laird, M. F. (2023). Energetic costs of feeding in 12 species of small-bodied primates. *Philos. Trans. R. Soc. B* 378, 20220553. 10.1098/rstb.2022.0553 PMC10577031 37839441

[JEB251950C71] Ward, S. R., Winters, T. M., O'Connor, S. M. and Lieber, R. L. (2020). Non-linear scaling of passive mechanical properties in fibers, bundles, fascicles and whole rabbit muscles. *Front. Physiol.* 11, 211. 10.3389/fphys.2020.00211 32265730 PMC7098999

[JEB251950C72] Winters, T. M., Takahashi, M., Lieber, R. L. and Ward, S. R. (2011). Whole muscle length-tension relationships are accurately modeled as scaled sarcomeres in rabbit hindlimb muscles. *J. Biomech.* 44, 109-115. 10.1016/j.jbiomech.2010.08.033 20889156 PMC3003754

[JEB251950C73] Yemm, R. (1976). *The Role of Tissue Elasticity in the Control of Mandibular Resting Posture*. Mastication.

[JEB251950C74] Zajac, F. E. (1989). Muscle and tendon: properties, models, scaling, and application to biomechanics and motor control. *Crit. Rev. Biomed. Eng.* 17, 359-410. 2676342

