# Supplementary material for: Bite force–gape curves and passive tension costs in Macaca mulatta
Source: J Exp Biol. 2026 May 5;229(9):jeb251950. doi: 10.1242/jeb.251950 (PMC13200732; doi:10.1242/jeb.251950)
Supplement: Supplementary information [file jexbio-229-251950-s1.pdf]

A.

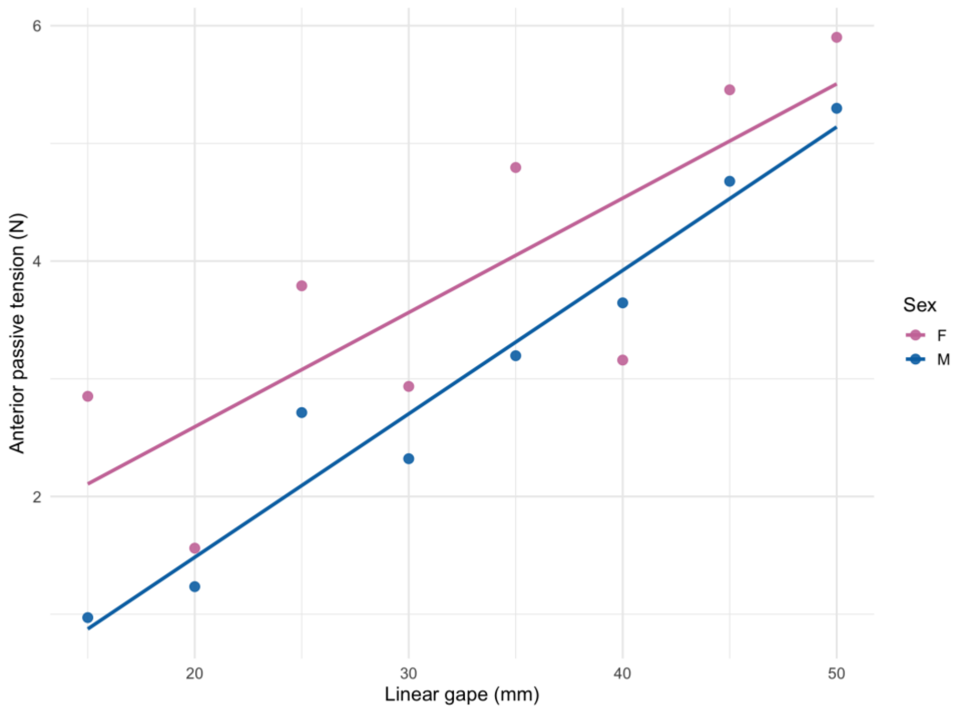

B.

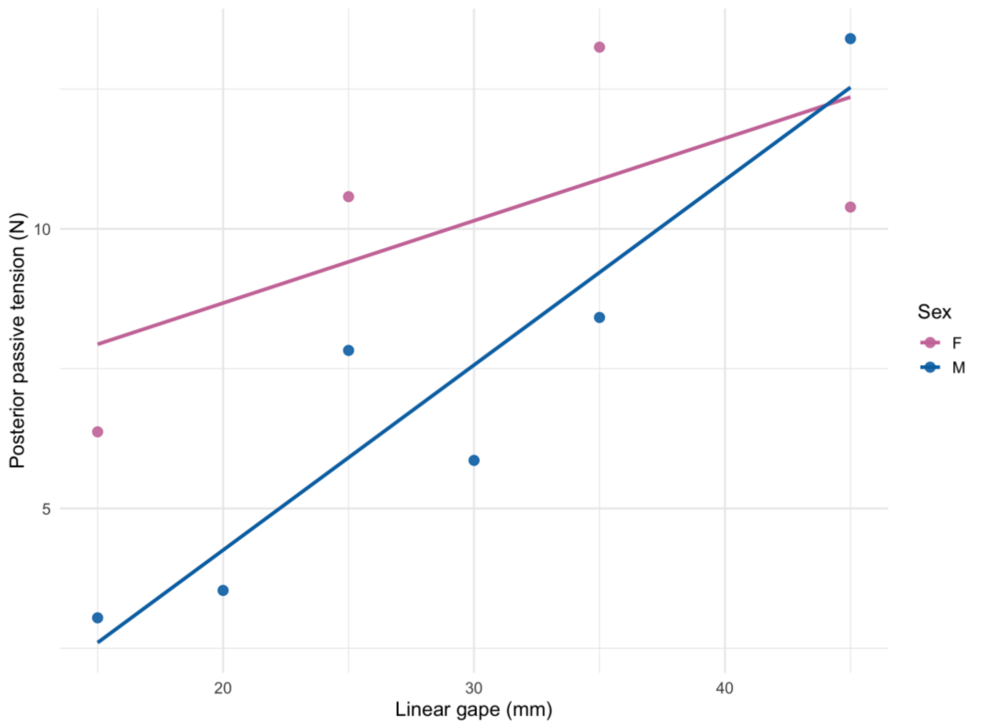

Fig. S1. Differences in maximum passive forces between males and females at I_1 (**A**) and M_1 (**B**). Females had significantly higher passive forces in anterior bite points for all gapes.

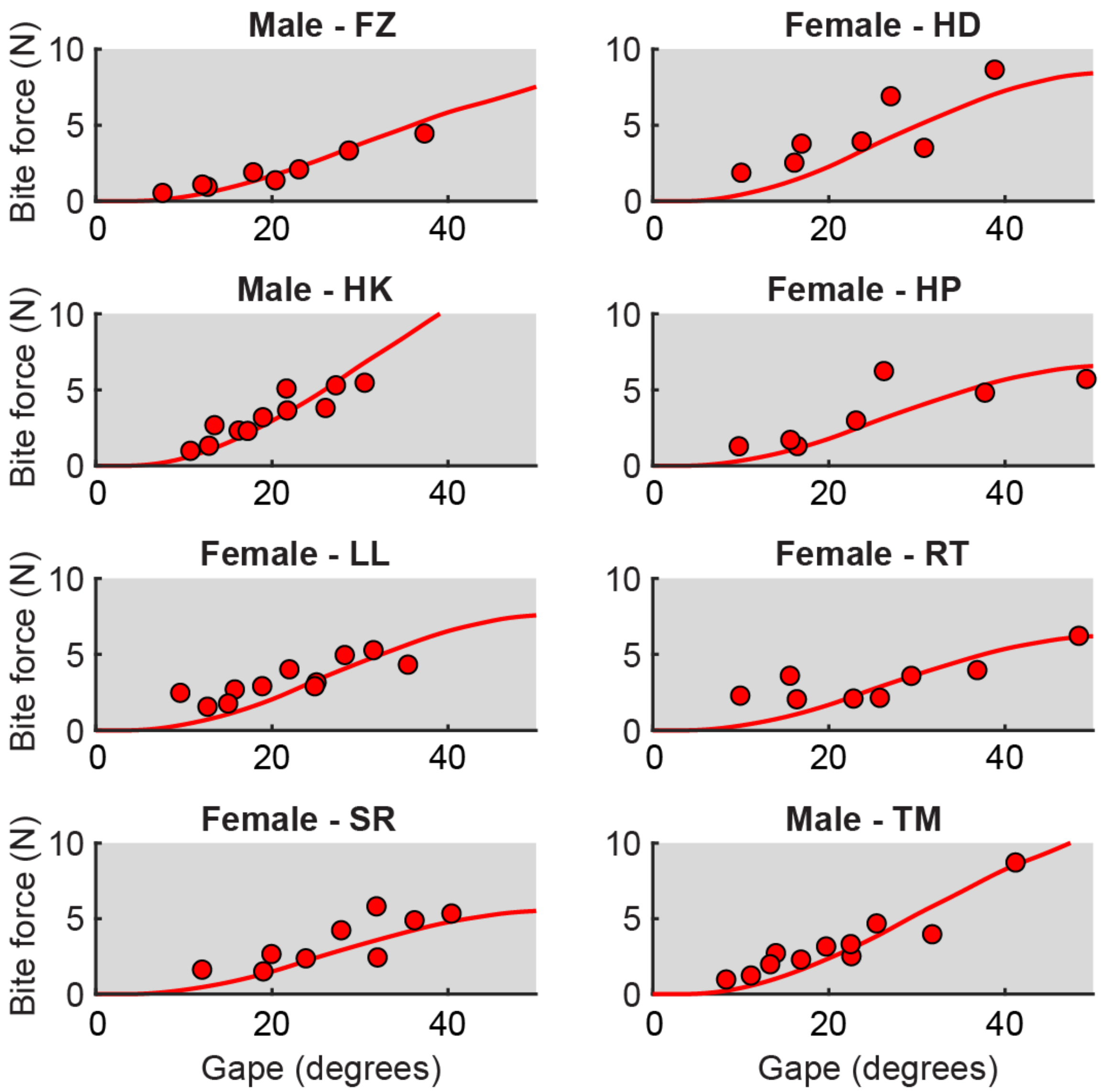

Fig. S2. Comparison of experimentally collected passive bite force data on eight anesthetized individuals. The red line shows the predicted passive bite force data for a given gape angle from muscle models optimized for each individual.

Journal of Experimental Biology • Supplementary information

Table S1. Mechanical and physical properties of walnut and brazil nut shells.

Food type	*n*	Maximum length (average; mm)	Maximum breadth (average; mm)	Minimum breadth (average; mm)	Force-to-fracture (average; N)[1]
Brazil nut	6	46.75	22.68	12.23	614.46
Walnut	6	35.13	33.10	31.08	233.39

[1]Force-to-fracture data from Laird et al. (2023)

Table S2. Location of catastrophic fracture of shelled nuts by LL and HK.

Monkey ID	Sex	Nut type	Food #	Location of initial bite	Location of fracture
HK	M	Brazil nut	1	posterior	posterior
HK	M	Brazil nut	2	anterior	anterior
HK	M	Brazil nut	3	anterior	posterior
HK	M	Brazil nut	4	anterior	posterior
HK	M	Brazil nut	5	anterior	anterior
LL	F	Brazil nut	1	posterior	posterior
LL	F	Brazil nut	2	posterior	posterior
LL	F	Brazil nut	3	anterior	posterior
LL	F	Brazil nut	4	posterior	posterior
HK	M	Walnut	1	anterior	anterior
HK	M	Walnut	2	anterior	posterior
HK	M	Walnut	3	anterior	anterior
HK	M	Walnut	4	anterior	anterior
HK	M	Walnut	5	anterior	anterior
HK	M	Walnut	6	anterior	anterior

LL	F	Walnut	X	Attempted to fracture (briefly pouched); discarded.	
LL	F	Walnut	X	Attempted to fracture (briefly pouched); discarded.	
LL	F	Walnut	1	posterior	posterior
LL	F	Walnut	2	posterior	posterior
LL	F	Walnut	X	Attempted to fracture (briefly pouched); discarded.	
LL	F	Walnut	3	posterior	posterior
LL	F	Walnut	4	posterior	posterior

[1]Catastrophic fractures were easily observable whereas the initial microfractures were not consistently observable (on the food item) or audible (due to the noise by other macaques in the room).

Journal of Experimental Biology • Supplementary information

Table S3. Results from LME models testing differences between passive forces measured on the anterior and posterior dentition, and between males and females on the anterior and position dentition.

**All individuals-Linear gape-Anterior and Posterior**					
	**Estimate**	**Std. Error**	**df**	**t value**	**p.value**
(Intercept)	0.21491	0.75001	49.28609	0.287	0.776
LinearGape	0.08846	0.01996	61.38592	4.431	3.94E-05
LocationPosterior	-0.05405	1.01468	61.41321	-0.053	0.958
LinearGape:Location Posterior	0.14214	0.03339	62.04977	4.257	7.13E-05
contrast	estimate	SE	df	t.ratio	p.value
Anterior-Posterior	-4.17	0.356	62.3	-11.706	<.0001
**All individuals-Angular gape-Anterior and Posterior**					
	**Estimate**	**Std. Error**	**df**	**t value**	**p.value**
(Intercept)	0.37725	0.685	46.36551	0.551	0.584
LinearGape	0.06904	0.01514	65.86135	4.561	2.27E-05
LocationPosterior	0.15172	0.96236	62.66896	0.158	0.875
LinearGape:Location	0.11624	0.02684	63.93886	4.331	5.34E-05

Posterior					
contrast	estimate	SE	df	t.ratio	p.value
Anterior-Posterior	-4.28	0.37	62.7	-11.571	<.0001
**Passive forces-Linear gape-Anterior only**					
	**Estimate**	**Std. Error**	**df**	**t value**	**p.value**
(Intercept)	0.77542	0.41803	36.54481	1.855	0.0717
LinearGape	0.08132	0.01184	34.43693	6.871	6.11E-08
SexM	-1.50633	0.69527	36.89506	-2.167	0.0368
LinearGape:SexM	0.01787	0.01976	33.6759	0.904	0.3724
contrast	estimate	SE	df	t.ratio	p.value
F-M	0.948	0.33	5.58	2.874	0.0307
**Passive forces-Angular gape-Anterior only**					
	**Estimate**	**Std. Error**	**df**	**t value**	**p.value**
(Intercept)	1.018279	0.367189	29.30161	2.773	0.00956
LinearGape	0.056762	0.007842	38.86831	7.238	1.03E-08

SexM	-1.72906	0.634353	34.46259	-2.726	0.01001
LinearGape:SexM	0.035582	0.015844	35.21285	2.246	0.03109
contrast	estimate	SE	df	t.ratio	p.value
F-M	0.376	0.317	5.87	1.184	0.2822
**Passive forces-Linear gape-Posterior only**					
	**Estimate**	**Std. Error**	**df**	**t value**	**p.value**
(Intercept)	1.41292	1.56399	24.91397	0.903	0.374961
LinearGape	0.21456	0.05126	20.92949	4.186	0.000419
SexM	-3.20662	2.42353	24.62706	-1.323	0.197951
LinearGape:SexM	0.04248	0.07611	20.34013	0.558	0.582878
contrast	estimate	SE	df	t.ratio	p.value
F-M	2.04	1.24	5.47	1.651	0.154
**Passive forces- Angular gape-Posterior only**					
	**Estimate**	**Std. Error**	**df**	**t value**	**p.value**
(Intercept)	2.02824	1.55555	24.25873	1.304	0.204515

Journal of Experimental Biology • Supplementary information

angulargape	0.15328	0.04009	21.42948	3.824	0.000961
SexM	-3.85587	2.43064	23.93128	-1.586	0.125784
angulargape:SexM	0.09023	0.06616	20.32756	1.364	0.187539
contrast	estimate	SE	df	t.ratio	p.value
F-M	0.985	1.3	5.52	0.757	0.4804

Table S4. Confidence intervals for the relationships between anterior and posterior passive forces and gape.

**All animals: Anterior Passive forces**					
**Gape**	**emmean**	**SE**	**df**	**lower.CL**	**upper.CL**
**15mm**	1.77	0.446	1.41	**-1.156**	**4.69**
**20mm**	1.06	0.507	2.44	**-0.786**	**2.91**
**25mm**	2.5	0.438	1.33	**-0.665**	**5.66**

Journal of Experimental Biology • Supplementary information

**30mm**	2.27	0.482	1.97	0.172	4.37
**35mm**	3.33	0.438	1.33	0.171	6.49
**40mm**	2.73	0.482	1.97	0.636	4.83
**45mm**	4.07	0.454	1.53	1.414	6.73
**50mm**	5.02	0.51	2.44	3.163	6.87
**All animals: Posterior Passive forces**					
**Gape**	**emmean**	**SE**	**df**	**lower.CL**	**upper.CL**
**15mm**	3.17	1.15	1.91	**-1.972**	**8.32**
**20mm**	3.64	2.49	15.98	**-1.638**	**8.91**
**25mm**	6.67	1.15	1.91	1.527	11.82
**30mm**	5.96	2.49	15.98	0.687	11.24
**35mm**	7.81	1.18	2.21	3.169	12.45
**45mm**	10.43	1.4	4.17	6.599	14.27

Journal of Experimental Biology • Supplementary information

Table S5. Passive component parameters optimized from passive bite force data.

ID	Sex	Passive parameters		
		ΔL_p	cf_p	R^2 *adjusted*
FZ	M	0.28	0.04	0.82
HK	M	0.24	0.09	0.58
TM	M	0.28	0.08	0.74
HD	F	0.34	0.09	0.28
HP	F	0.38	0.06	0.16
LL	F	0.31	0.06	0.27
RT	F	0.37	0.05	0.31
SR	F	0.36	0.10	0.18
